# Altered Expression of Two Small Secreted Proteins (*ssp4* and *ssp6*) Affects the Degradation of a Natural Lignocellulosic Substrate by *Pleurotus ostreatus*

**DOI:** 10.3390/ijms242316828

**Published:** 2023-11-27

**Authors:** Oded Yarden, Jiwei Zhang, Dor Marcus, Chunoti Changwal, Sameer J. Mabjeesh, Anna Lipzen, Yu Zhang, Emily Savage, Vivian Ng, Igor V. Grigoriev, Yitzhak Hadar

**Affiliations:** 1 Department of Plant Pathology and Microbiology, The Robert H. Smith Faculty of Agriculture, Food and Environment, The Hebrew University of Jerusalem, Rehovot 7610001, Israel; dormarcus18@gmail.com (D.M.); chunoti.changwal@mail.huji.ac.il (C.C.); yitzhak.hadar@mail.huji.ac.il (Y.H.); 2 Department of Bioproducts and Biosystems Engineering, University of Minnesota, Saint Paul, MN 55108, USA; zhan3437@umn.edu; 3 Department of Animal Sciences, The Robert H. Smith Faculty of Agriculture, Food and Environment, The Hebrew University of Jerusalem, Rehovot 7610001, Israel; sameer.mabjeesh@mail.huji.ac.il; 4 DOE Joint Genome Institute, Lawrence Berkeley National Laboratory, Berkeley, CA 94720, USA; alipzen@lbl.gov (A.L.); yzhang13@lbl.gov (Y.Z.); emilysavage@lbl.gov (E.S.); vng@lbl.gov (V.N.); ivgrigoriev@lbl.gov (I.V.G.); 5 Department of Plant and Microbial Biology, University of California Berkeley, Berkeley, CA 94720, USA

**Keywords:** white rot fungus, small secreted protein, lignin, pectinase, plant biomass degradation, SSP

## Abstract

*Pleurotus ostreatus* is a white-rot fungus that can degrade lignin in a preferential manner using a variety of extracellular enzymes, including manganese and versatile peroxidases (encoded by the *vp1-3* and *mnp1-6* genes, respectively). This fungus also secretes a family of structurally related small secreted proteins (SSPs) encoded by the *ssp1-6* genes. Using RNA sequencing (RNA-seq), we determined that *ssp4* and *ssp6* are the predominant members of this gene family that were expressed by *P. ostreatus* during the first three weeks of growth on wheat straw. Downregulation of *ssp4* in a strain harboring an *ssp* RNAi construct (KD*ssp1*) was then confirmed, which, along with an increase in *ssp6* transcript levels, coincided with reduced lignin degradation and the downregulation of *vp2* and *mnp1*. In contrast, we observed an increase in the expression of genes related to pectin and side-chain hemicellulose degradation, which was accompanied by an increase in extracellular pectin-degrading capacity. Genome-wide comparisons between the KD*ssp1* and the wild-type strains demonstrated that *ssp* silencing conferred accumulated changes in gene expression at the advanced cultivation stages in an adaptive rather than an inductive mode of transcriptional response. Based on co-expression networking, crucial gene modules were identified and linked to the *ssp* knockdown genotype at different cultivation times. Based on these data, as well as previous studies, we propose that *P. ostreatus* SSPs have potential roles in modulating the lignocellulolytic and pectinolytic systems, as well as a variety of fundamental biological processes related to fungal growth and development.

## 1. Introduction

The genus *Pleurotus* comprises cultivated, edible, ligninolytic mushrooms with a wide array of biotechnological and environmental applications [1,2]. *P. ostreatus* represents one of the most well-studied model species in this genus, and as a white-rot fungus (WRF), it is able to grow on a variety of lignocellulosic substrates and degrade the lignin in a preferential manner [3,4]. It also has attested capabilities of degrading natural and xenobiotic recalcitrant aromatic compounds, and this is largely attributed to the activity of its non-specific oxidative enzymatic systems, which are composed of versatile ligninolytic enzymes including extracellular laccases, versatile peroxidases, and manganese peroxidases [5,6,7]; H_2_O_2_-producing auxiliary oxidases; and intracellular oxidizing enzymes such as a variety of P450 monooxygenases [2]. The fact that *P. ostreatus* is amenable to genetic manipulations, including the versatile toolkits for conducting gene silencing, overexpression, and disruption [8,9,10], provides a feasible platform to elucidate the mechanistic aspects of plant biomass substrate’s conversion.

Small Secreted Proteins (SSPs) are an arbitrary class of proteins that are characteristically defined as being fewer than 300 amino acids in length and containing a signal peptide. Commonly, 10–20% of fungal secretomes, including that of *P. ostreatus*, are SSPs, regardless of their lifestyle and the nature of the substrates they utilize [11,12]. While some of the functions of fungal SSPs are known, including effector-like proteins, hydrophobins, and cerato-platanins [13,14,15], the precise functions of many SSPs have not yet been fully elucidated [16]. This knowledge gap is particularly evident in saprophytic species, which include white rot fungi.

The genome of the monokaryotic *P. ostreatus* strain PC9 encodes over 500 SSPs, approximately 30% of which are unique to this species [16]. Characterization of several of these SSPs has been conducted by analyzing their expression profiles, which showed three members of a subgroup of *P. ostreatus* SSPs (65712, 46202, and 91630, designated *ssp1*, *ssp2,* and *ssp3*, respectively; Protein IDs were retrieved from JGI’s *Pleurotus ostreatus* PC9 v1.0 genome) and presented very low expression in the nutritional liquid medium (i.e., Glucose-Peptone medium), while their expression was significantly elevated with the supplement of 5-hydroxymethylfurfural (5-HMF; a common toxic compound generated during the pretreatment of cellulosic biomass), indicating that SSPs might play certain roles in fungal response to 5-HMF [17]. Similarly, another study reported that *ssp1* was slightly expressed in *P. ostreatus* in both static and shaken cultures in a sucrose–malt extract–yeast extract (SMY) liquid medium [18]. In addition to responding to 5-HMF, it was also found that *ssp* genes’ expressions were likely correlated with the activities of the ligninolytic system in *P. ostreatus* [17]. This potential relevance of SSPs to lignin degradation has also been documented by Wu et al. [19], who reported that the *ssp4* gene was the most highly expressed on rice straw and beech wood sawdust substrates in both the wild type and mutants exhibiting an impaired ligninolytic system. Furthermore, it found that the disruption of *gat1*, a putative transcriptional regulator involved in regulating ligninolytic genes’ expression on beech wood sawdust, significantly upregulated (hundreds to thousands of folds) the expression of another *ssp* gene—*ssp1* [19,20]. The data described above provide accumulating evidence for the presence of a link between *ssp* expression and that of some of the lignocellulolytic machinery. However, the genetic association of *ssp*s to lignocellulose degradation is still not known.

To study the functions of SSPs in laboratory media, we employed RNAi to target an SSP subgroup composed of the *ssp1-6* genes in *P. ostreatus* [17,21]. In those studies, the assumption was that RNAi-based expression of *ssp1* would result in co-silencing of the six genes. The analyses on mutants showed that the SSP knockdown (KD*ssp1*) affected the overall repertoire of fungal secretome, metabolism, and development, as reflected by a quicker transition from tropophase to idiophase in the KD*ssp1* mutant than that occurring in the wild-type strain [17,21]. The data obtained also demonstrated that the KD*ssp1* strain exhibited impaired protein synthesis of aryl-alcohol oxidase, versatile peroxidase, and several glycoside hydrolases (e.g., GH5). Although those studies clearly indicated that SSPs have a functional role in the saprotrophic lifestyle of *P. ostreatus*, limitations of analytical methods have been hindering us from deciphering the functions of SSP in regulating lignocellulose degradation. First, the liquid cultural conditions employed in previous studies have restricted us from exploring the fungal response to its natural substrate environments. In other fungi such as *Aspergillus niger* [22] and *Phanerochaete chrysosporium* [23], it has been clearly demonstrated that the transcriptional response can vary immensely between growth in liquid- versus solid-culture conditions. Second, the use of non-lignocellulosic substrates may not reflect the natural biomass resource used for living the saprotrophic life in *P. ostreatus*, thereby hindering us from exploring its lignocellulose-degrading machinery. Third, the previous analyses, which were based on measuring the secretome and single-gene expression, cannot provide sufficient information for the systematic understanding of SSP functions.

Here, we grew the *P. ostreatus* wild type and the KD*ssp1* mutant on agricultural feedstock, wheat straw, for solid-state fermentation, followed by characterization of the fate of components of the lignocellulosic substrate, measuring the temporal transcriptome, and conducting co-expression network analysis to link genes’ functions to the two genotypes used. Using RNA-seq to resolve the similar sequences of orthologue genes, we determined that only *ssp4* and *ssp6* are expressed during the first three weeks in *P. ostreatus* growing on wheat straw. We found that the downregulation of *ssp4* in the KD*ssp1* strain was chronologically synchronized with reduced lignin degradation and the decreased expression of *vp2* and *mnp1* that encode significant components of the lignocellulose-degrading machinery. Further RNA-seq analysis revealed that this was also accompanied by a significant increase in the expression of genes related to pectin and side-chain hemicellulose degradation. Using co-expression network analysis, gene modules that responded to *ssp* knock-down, along with their potential biological functions, were identified. Taken together, we propose that while some *ssp*s are clearly involved in converting lignocellulose, other structurally related *ssp*s are likely to have other roles during growth, development, and natural substrate utilization by *P. ostreatus*.

## 2. Results

### 2.1. Members of the Six-Gene ssp Family Exhibit Diverse Expression Patterns during Growth of Pleurotus ostreatus on Wheat Straw 

**Figure 1:**
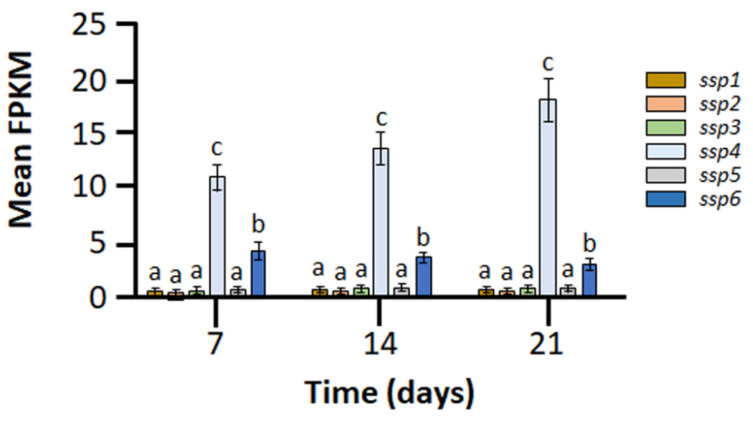
Expression levels of the 6-gene *ssp* family in *P. ostreatus* PC9 cultured on wheat straw, as determined by RNA sequencing to measure the fragments per kilobase of transcript per million mapped reads (FPKM) value. Bars indicate mean values and standard errors on the basis of five replicates. Letters indicate significant differences as determined by one-way ANOVA (*p* < 0.05).

RNA sequencing was carried out in order to determine the changes in the transcriptional profile of the *P. ostreatus* wild type and, as described below, the KD*ssp1* strain, cultured on wheat straw as a natural lignocellulosic substrate. While the six-gene family members share a significant extent of structural similarity, their expression patterns throughout the time course of this experiment varied immensely. Among these, *ssp1*, *ssp2*, *ssp3,* and *ssp5* showed marginal expression throughout the entire 3-week sampling period (Figure 1), while the expression of *ssp6* was significantly higher than the detection threshold and was constant throughout the experiment. *ssp4* stood out as the most highly expressed family member, whose expression levels were not only the highest but also increased over the time course of the experiment. Based on these data, we concluded that the transcriptional patterns of the genes in this *ssp* subgroup are diverse during fungal proliferation on a natural substrate and that even though *ssp4* and *ssp6* are significantly expressed, they do not share a temporal regulatory pattern, indicating they may have different roles.

### 2.2. Changes in the Expression of Two ssp Genes during Growth on Wheat Straw Impose a Change in Lignin Degradation 

**Figure 2:**
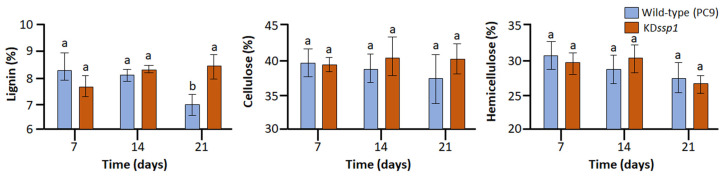
Lignocellulose contents of wheat straw after degradation by either the wild-type PC9 (blue) or KD*ssp1* (orange) strains of *P. ostreatus*. The fractions presented were calculated based on neutral detergent fiber (NDF), acid detergent fiber (ADF), and acid detergent lignin (ADL) analyses. Bars indicate standard errors (*n* = 5). When relevant, letters within drawings indicate significant differences as determined by one-way ANOVA (*p* < 0.05).

To determine whether the KD*ssp1* mutant was affected in its capability to degrade wheat straw, the two strains were cultured for 3 weeks on the lignocellulosic substrate. We analyzed the lignin, cellulose, and hemicellulose contents using the Van Soest method [24], a standard procedure used to determine nutrient content and availability to farm animal digestion. While the cellulose and hemicellulose contents of the substrates were not differentially affected by the growth of the wild type and the KD*ssp1* strain, at the end of the 3-week growth period, lignin content was about 20% higher in the substrate on which the mutant was cultured on (Figure 2). It is noteworthy that a major decrease in lignin content in PC9 was observed between the second and third weeks. 

**Figure 3:**
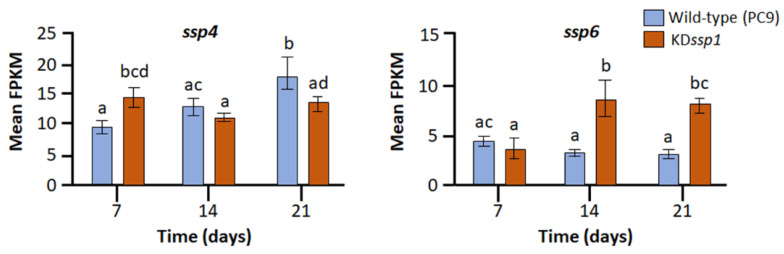
Expression levels of *P. ostreatus ssp4* and *ssp6* in the wild-type (blue) and KD*ssp1* (orange) strains grown on a wheat straw substrate. Bars indicate standard errors (*n* = 5). Letters within drawings indicate significant differences as determined by one-way ANOVA (*p* < 0.05).

Further analyses of the whole-genome expression profiles in the KD*ssp1* strain were employed in order to establish whether the presence of the *ssp1*-based RNAi construct had an effect on the expression of *ssp*s and additional genes that might be involved in lignin degradation. Although the KD*ssp1* mutant was designed to target all the *ssp1–6* subgroup member genes, as *ssp4* and *ssp6* were the most abundantly expressed *ssp*s during growth on the wheat straw substrate, we expected to observe the major effects of the RNAi construct on the transcript abundance of these genes. While *ssp* gene expression in the knockdown strain was not eliminated, at the advanced stage of growth (i.e., 21 days), the expression of *ssp4* was downregulated when compared to that of the wild type (Figure 3). Unexpectedly, the transcript abundance of *ssp6* was higher in the KD*ssp1* mutant. Revisiting the sequence of the RNAi construct indicated that it has a high degree of similarity to the DNA sequence of *ssp1–5*, but much less so to that of *ssp6*. The fact that the nucleotide sequence of *ssp6* did not meet the criterion of continuous identity along a stretch of 18–22 nucleotides (Table S2) makes this gene less likely to be subjected to RNAi-based silencing, as recently suggested by Chen et al. [25]. Furthermore, we did not find additional stretches of ≥18 nucleotide identities to the *ssp1*-based construct within the *P. ostreatus* genome, thus reducing the chances of additional genes being silenced. 

In addition, to test the potential multiple mapping issues of homologous genes, we reran the mapping and reads counting by two additional stringent parameter sets, which generated no differences (Table S3 and Figure S1) relative to using the default parameters described in the Section 4 (Materials and Methods). Our parameter set 1 included using new flags of -k 5 --score-min “L,0,−0.04”, which saves and reports up to five valid alignments but changes the minimum alignment score to −6 from −30, which is equivalent to 1 mismatch per 150 bp read at a high-confidence site or 2–3 mismatches at low-confidence sites. Gene assignment counting was then carried out with the “primary” hit for any multi-mapping reads. Also, parameter set 2 included gene assignment counting without allowing any reads that mapped to multiple locations in the genome. Thus, based on the analysis of lignocellulosic residues in the culture substrate, along with our RNA-seq results, it was evident that impaired expression of *ssp4* and increased expression of *ssp*6 in the KD*ssp1* mutant grown on wheat straw was chronologically linked with reduced lignin degradation.

### 2.3. Expression of vp2 and mnp1, Components of the Lignin Degrading Machinery, Is Compromised in the KDssp1 Strain 

**Figure 4:**
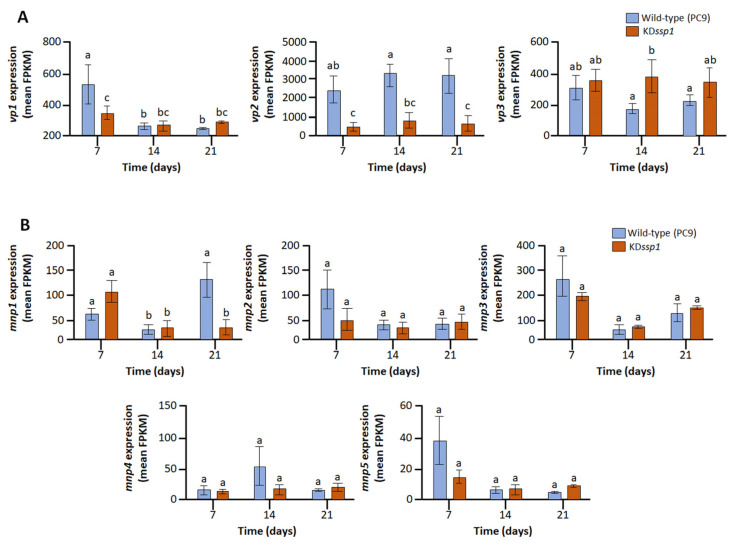
The effects of the reduced expression of *ssp4* on transcript abundance (along with the increase in ssp6 transcription) on components of the ligninolytic machinery in *P. ostreatus* wild type (blue) and the KD*ssp1* strain (orange) cultured on a wheat straw substrate, as determined by RNA sequencing. The expression of versatile peroxidases (*vp1*-116738, *vp2*-60432, and *vp3*-123383) (**A**) and manganese peroxidases (*mnp1*-115087, *mnp2*-61491, *mnp3*-51690, *mnp4*-121638, and *mnp5*-52120) (**B**) are presented. Bars indicate standard errors (*n* = 5). Letters within drawings indicate significant differences as determined by one-way ANOVA (*p* < 0.05).

As *P. ostreatus* SSPs have no known catalytic activity, yet their impaired transcription affected lignin degradation, we examined the fungus’ transcriptional profile for possible changes in the transcript abundance of genes encoding proteins of the lignin-degrading machinery. We found that the most abundantly transcribed VP-encoding gene was *vp2* (60432), whose transcript abundance was several folds higher than the other two *vp*s in the wild type. The transcription of *vp2* was the most pronouncedly affected in the mutant strain, and its transcript abundance was only about 15% of that observed in the wild type (Figure 4A). Interestingly, and in a manner different from that observed for many other genes whose transcript levels had been affected, the reduced levels of *vp2* transcripts were evident even after one week of culturing on the wheat straw. While five of the six *P. ostreatus mnp* genes were expressed throughout the experiment, only *mnp1* (115087) exhibited significantly impaired expression in the mutant. Specifically, at the third-week sampling point, the abundance of *mnp2* (61491) transcripts in the KD*ssp1* strain was less than a third of that observed in the wild type (Figure 4B). 

**Figure 5:**
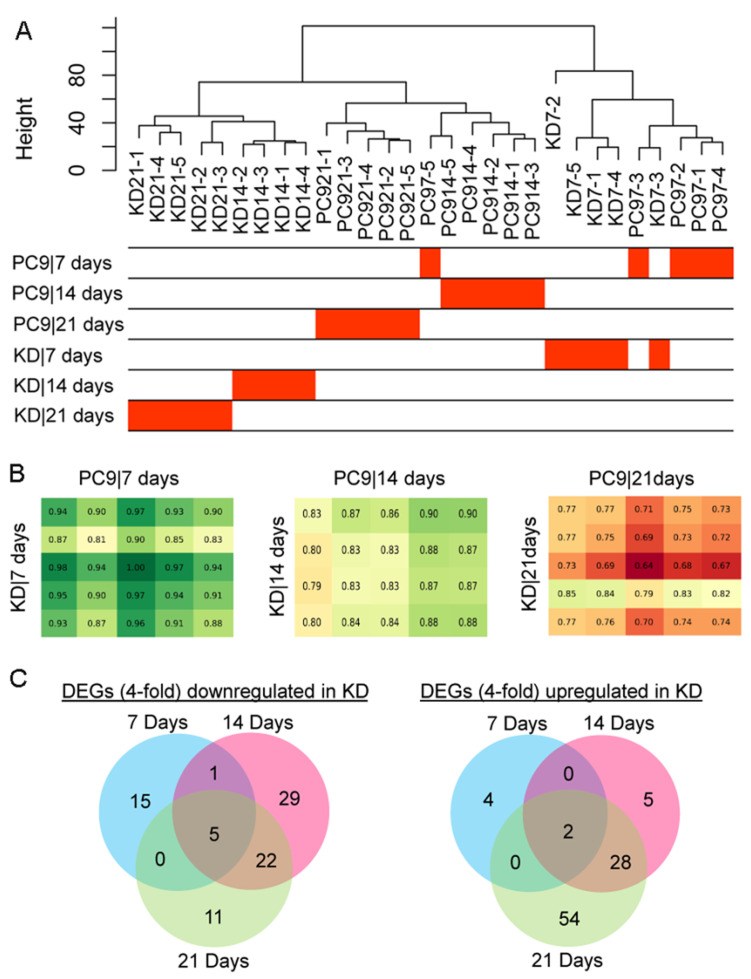
All gene-expression patterns in the *Pleurotus ostreatus* KD*ssp1* mutant versus the wild-type PC9 cultured on wheat straw over a three-week period. Sample clustering based on the whole-genome expression shows the progressively accumulated transcriptional changes in response to SSP knock-down, with evident genotype-dependent sample segregation at the advanced cultivation stages (**A**). This is also reflected by the pairwise (KD*ssp1* vs. PC9) correlation analyses across the time-scale (**B**), in which the green and red colors indicate the similarity (i.e., higher correlation values) and difference between samples, respectively. The columns and rows of the correlation matrix represent the bioreplicates of the corresponding tests. Prior to the analysis, those constitutively lowly expressed genes were removed, thus keeping 9742 out of 12204 whole genes to maintain the expression levels of FPKM > 1 in all samples. Sample tree was built using the “average” method. (**C**) The number of significantly differentially expressed genes (DEG; fold change > 4 and FDR < 0.05) that were either upregulated or downregulated in the KD*ssp1* mutant were then highlighted in Venn diagram for each cultivation time.

Whole-genome transcription profiles were progressively altered in the KD*ssp1* mutant. In addition to following the expression patterns of the ligninolytic machinery, we analyzed the overall changes in the transcriptional profile of KD*ssp1*, relative to the wild type, when grown on wheat straw over time. The results of sample clustering and correlation analysis established the presence of distinct differences between the KD*ssp1* and wild-type genotypes and that the genotype became a dominant factor in segregating samples at the advanced cultivation stages (i.e., 14 and 21 days). Hence, we observed that the differences were in line with impaired *ssp* expression progressively accumulated in the KD*ssp1* mutant (Figure 5A,B). Based on these observations, our approach was then focused on conducting pairwise comparisons of the wild type and the mutant (PC9 vs. KD*ssp1*), which allowed us to quarry the significantly differentially expressed genes (DEGs; fold change > 4 and FDR < 0.05) in the KD*ssp1* mutant at different cultivation times (Figure 5C). In line with the progressively accumulated changes, more DEGs were identified for the advanced cultivation stages (Figure 5C). Specifically, while only 27 DEGs were identified when analyzing the 7-day cultivation sampling time point, this number substantially increased to 92 and 122 DEGs after 14 and 21 days, respectively. Overall, these results are indicative of the presence of an adaptive, rather than a rapidly induced, pattern of a transcriptional response to altered ssp transcript levels. 

### 2.4. Identification of Key Differentially Expressed Genes (DEGs) Associated with the Knockdown of ssps

The DEGs in KD*ssp1*, relative to the wild type, that were found to overlap at both advanced cultivation times are listed in Table 1. Among these, 27 DEGs were significantly downregulated, while 30 were upregulated in KD*ssp1* (Table 1). Analysis of the downregulated DEGs showed that no GO (Gene Ontology) functions were significantly enriched (Table S4). These downregulated DEGs included those encoding a GMC oxidoreductase (88910; AA3 family) and a galactose oxidase-like protein (99670; AA5 family) that may function as the axillary, H_2_O_2_-generating enzymes, providing an electron acceptor to aid the lignin oxidation catalyzed by VP2 (60432) that was also identified as a significantly downregulated DEG in KD*ssp1*. In addition to the genes related to ligninolytic functions, there are also downregulated DEGs that are associated with basic cellular structure, signaling, and metabolism functions, as well as unknown proteins. Notably, the expression of a gene encoding a hemopexin-like protein (133592) decreased by almost 500-fold from the FPKM values of 2000–6000 in the wild type to 5–130 in the KDssp1 mutant (Table S1). It has been found that the hemopexin-like protein may play roles in iron recycling or as a metalloproteinase [26,27], but what specific function it may have in relation to SSPs remains to be determined. 

**Figure 6:**
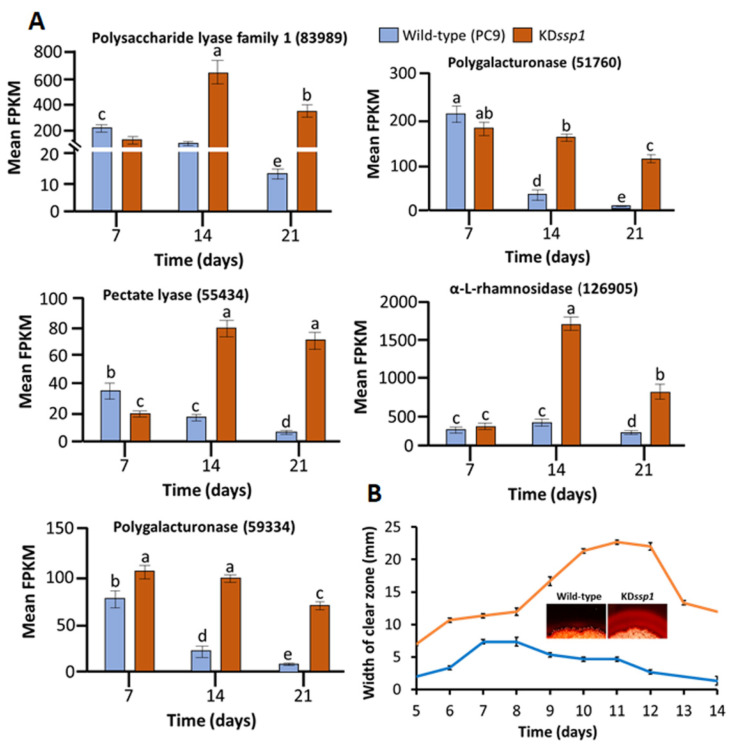
The effects of the reduced expression of *ssp4* (along with the increased levels of *ssp6* transcription) on transcript abundance of two pectin lyases, 83989 and 55434 (polysaccharide lyase family 1; PL1; pectate lyase), two endo-polygalacturonase (59334 and 51760; GH28), and one alpha-L-rhamnosidase (126905; GH37; trehalase) in *P. ostreatus* wild type (blue) and the KD*ssp1* strain (orange) cultured on a wheat straw substrate, as determined by RNA sequencing (**A**). Pectin-degradation capacity of the *P. ostreatus* strains grown on pectin-rich media, as determined in 3 experiments, by visualization of pectin degradation zones indicated by Congo Red. Insert depicts comparative pectin degradation zones at day 12 as produced by the respective strains (**B**). Letters within drawings indicate significant differences as determined by one-way ANOVA (*p* < 0.05). Bars indicate standard errors (*n* = 5 and *n* = 3 for (**A**) and (**B**), respectively).

In contrast, many GO functions were significantly over-represented in the upregulated DEGs, which included those associated with, for example, “cell wall organization or biogenesis”, “hydrolase activity acting on glycosyl bonds”, and “pectin catabolic processes” (Table S4). In line with the GO enrichment analysis, 5 out of the 30 upregulated DEGs were genes encoding pectin-degrading enzymes, including 3 pectin lyases (83989, 55434, 59334), 1 endo-polygalacturonase (51760; GH28), and 1 alpha-L-rhamnosidase (126905; GH37) (Table 1; Figure 6A). As we were intrigued by the dramatic increase in the expression of pectin lyases, which paralleled the decrease in the expression of some genes involved in lignin degradation, we analyzed the capacity of the two strains to degrade pectin in pectin agar medium (PAM). Indeed, and as implied by the RNA-seq results, pectin-degrading capacity in the KD*ssp1* strain culture was significantly higher than in the wild type, as indicated by the width of the pectin-clear zones produced by the strains over the time course of the experiment (Figure 6B). Three DEGs were highlighted as harboring side-chain hemicellulose-degrading functions, including an alpha-galactosidase (89942; GH27), an alpha-N-arabinofuranosidase (97623; GH43), and a GH32 enzyme (62766). Two DEGs encoding the GH24 and GH25 lysozymes (96680 and 117545) were associated with the functions of fungal cell wall construction. In addition, two hypothetical cerato-platanin proteins were also identified as DEGs that may bind to chitin and *N*-acetylglucosamine oligosaccharides and are involved in the biogenesis of fungal cell wall structures [13]. 

In addition to the DEGs with CAZyme functions, two carotenoid ester lipase precursors (116339 and 126566) were significantly upregulated in the KD*ssp1* mutant, which indicated that SSPs may be involved in regulating the synthesis of carotenoids or related pigments [28]. 

Taken together, the knockdown of the *ssp*s in *P. ostreatus* has positively influenced the expressions of genes associated with CAZymes involved in the degradation of substrates like pectin, hemicellulose, and fungal cell walls. In contrast, the ligninolytic genes were more likely negatively influenced by the *ssp* knockdown, implying that SSPs might play opposing roles in regulating the two distinct degradative systems of lignin and polysaccharides.

### 2.5. Co-Expression Network Analysis Suggests the Roles SSPs May Have in Regulating Lignocellulose Degradation and Other Fungal Processes 

**Figure 7:**
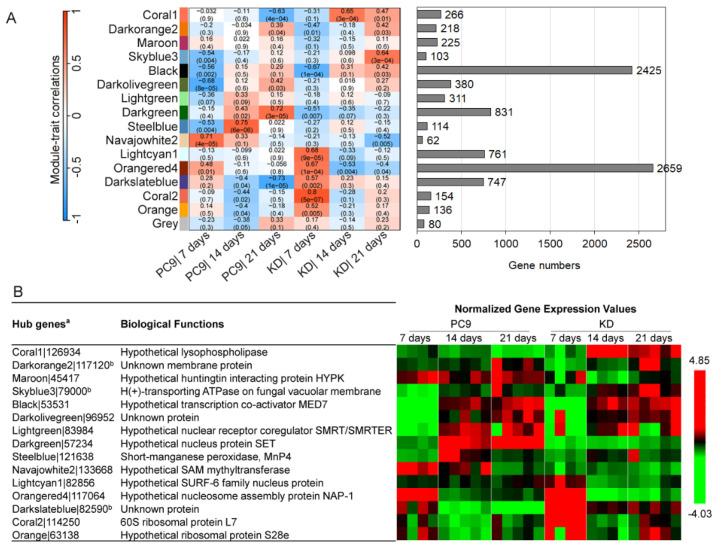
Genome-wide co-expression networking identified 16 gene modules that play roles in response to downregulation of *ssp*s. (**A**) Sixteen gene modules were identified based on their co-expression patterns in the KD*ssp1* and PC9 strains cultured on wheat straw over a three-week period, and their association with the genotypes (on the **left**) at different times was calculated based on module eigengenes’ expression. The number of genes in each module is depicted on the **right**. (**B**) The hub genes at the central place of each module’s network are highlighted, along with their predicted biological functions and the normalized expression levels as both strains decay the wheat straw across the experimental time scale. Gene expression levels of each hub gene were normalized with the standard (0, 1) uniform distribution. ^a^ Protein IDs were retrieved from the genome annotation of Pleurotus ostreatus PC9 v1.0 at JGI database (https://mycocosm.jgi.doe.gov/PleosPC9_1/PleosPC9_1.home.html; accessed on 1 March 2023). ^b^ The existence of protein secretion signals predicted by SignalP 6.0.

**Figure 8:**
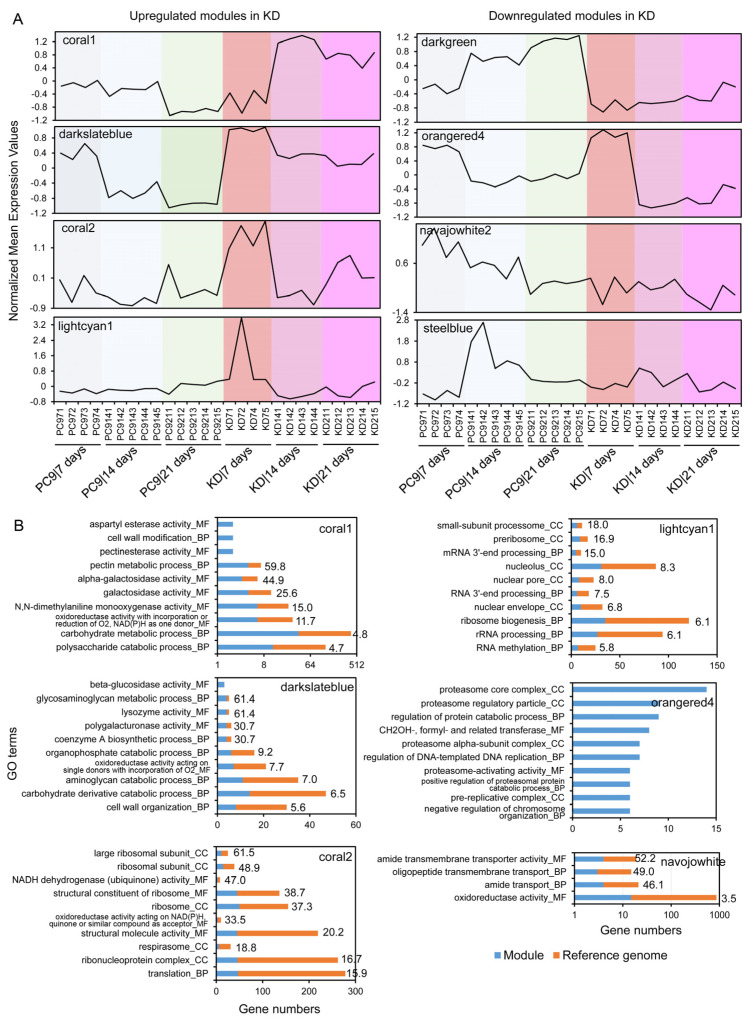
Biological functions of the crucial gene modules that are either positively or negatively responsive to the knockdown of *ssp*. (**A**) Expression patterns of gene modules in which the genes were either upregulated (e.g., coral1, darkslateblue, coral2, and lightcyan1) or downregulated (e.g., darkgreen, orangered4, navajowhite2, and steelblue) in the KD*ssp1* mutant relative to wild-type PC9 at different stages of cultivation on wheat straw. The expression pattern of each module was determined by calculating the averaged levels of gene expression. Gene expression data were transformed across samples with the (0, 1) normalization. (**B**) The top ten enriched functional GO categories of the selected gene modules. The numbers adjacent to the bars indicate the enrichment fold of the GO terms in the module of interest relative to the background levels in the reference genome. All the GO terms have FDR values of less than 0.05.

A WGCNA network analysis was used to identify possible functional modules on the basis of the gene co-expression patterns [29], and this identified 16 modules composed of 62–2659 genes (Figure 7A). Each module was then correlated with genotypes and cultivation times to dissect the key variable factors contributing to its expression pattern (Figure 8A). Hub genes, as well as their expression styles, were identified for each module, with 6 out of 15 hub genes predicted as nuclear or transcription-regulator functions, reflecting their core roles in the co-expression network (Figure 7B). Nonetheless, three of the hub genes have no functionally analyzed homologues (i.e., unknown proteins) in the database. In the coral1 module, the hub gene encodes a predicted esterase, indicative of its involvement in catalytic activity, yet its true function and potential substrates have yet to be determined. Similarly, functions of hub genes encoding H(+)-transporting ATPase on fungal vacuolar member (79000), short-manganese peroxidase MnP4 (121638), and SAM methyltransferase (133668) in the skyblue3, steelblue, and navajowhite2 modules, respectively, will also require further interrogation. 

Module–trait correlation analysis revealed four modules whose expressions were positively influenced in the KD*ssp1* mutant at either the advanced (coral1 and darkslateblue) or early cultivation stages (coral2 and lightcyan1), despite these modules’ temporal expression patterns in the wild-type strain being different (Figure 7A and Figure 8A). GO functional enrichment analysis revealed that modules coral1 and darkslateblue were over-represented by genes associated with pectin and hemicellulose degradation and cell wall construction (Figure 8B and Table S5), which is consistent with that revealed by the DEG analysis (Table 1). While not represented in the DEG analysis, we also found that genes in coral2 and lightcyan1 were upregulated in the KD*ssp1* mutant in response to the early cultivation stage and were enriched by functions associated with ribosome and protein synthesis, rRNA and mRNA processing, and nucleus formation (Figure 8B), indicating that the altered expression of *ssp* may have induced the fundamental requirements for recruiting the transcriptional and translational machinery of the fungal cells. 

Similarly, we identified four modules in which the genes’ expression levels were downregulated at different cultivation stages of the KD*ssp1* mutant (Figure 7A and Figure 8A). Among them, genes in the darkgreen module were clearly suppressed through all of the cultivation stages yet had no GO functions that were significantly over-represented (Table S5), suggesting that this downregulation effect in KD*ssp1* had affected a wide range of functions. Gene expression in orangered4 was not affected at 7 days but was greatly downregulated in KD*ssp1* as cultivation progressed. Surprisingly, more than 10% of GO terms of the entire genome (i.e., 582 out of 5045 GO terms; Table S5) were significantly enriched in this module, suggesting that a wide range of basic cellular functions have been downregulated due to the altered expression of *ssp*s. Gene functions related to the proteasome were highlighted in the orangered4 module as the significantly downregulated functions in KD*ssp1*, coinciding with the mutant’s needs of inducing protein synthesis revealed in upregulated modules coral2 and lightcyan1 (Figure 8B). The fact that components of the ubiquitin–proteasome system (UPS) were downregulated in the mutant may have significant implications regarding lignin degradation as in a different white rot fungus—*Trametes versicolor*—proteasomal degradation of intracellular proteins has been shown to be involved in the regulation of laccase activity in a nutrient-dependent manner [30]. Furthermore, as a variety of secondary metabolites have been shown to affect the UPS [31], it is possible that fungal SSPs may also play a role in either direct or secondary metabolite-mediated regulation of the UPS. In addition, the impaired SSP function also diminished the expression peaks at early or middle cultivation stages in the wild type, as seen in modules navajowhite2 and steelblue, respectively, although these modules only represented a limited number of genes (i.e., 62 and 114 genes). GO enrichment analysis indicated that these diminished functions were mostly related to roles involving amide and oligopeptide transport (Figure 8B). Taken together, the co-expression network analysis not only strengthened the SSP’s roles in regulating lignocellulolytic functions, but it also allowed us to discover, at a genome-wide level, its potential linkages to other fungal processes fundamental to fungal growth and development.

## 3. Discussion

While a significant part of the catalytic machinery involved in the bioconversion of lignocellulosic substrates by white rot fungi has been identified, the understanding of some of their regulatory aspects and “peripheral” components has yet to be completed. Information concerning the upstream, environmentally responsive factors such as transcriptional regulators is accumulating [32,33], and a potential functional link between SSPs and the expression of lignocellulose-degrading enzymes, including H_2_O_2_-producing enzymes, has been demonstrated [16,17,21]. Nonetheless, this has been, for the most part, studied in liquid cultures. Here, under solid-state fermentation, we determined the expression patterns of a subfamily of *P*. *ostreatus ssp*s and revealed that only two (*ssp4* and *ssp6*) out of the six members are expressed above threshold levels along the time course of growth on a natural wheat straw substrate. This finding is in agreement with previously published datatsets on other natural substrates, beech wood and rice straw, in which the biased effects of substrates on the lignocellulose-degrading machinery were investigated [19,32]. One explanation for the distinct *ssp* expression patterns could be the presence of partial or full redundancy among the genes and their products. On the other hand, this does not rule out the possibility of unique functions that each (or some) of the SSPs may have. For example, it has been shown that SSP1 was dramatically elevated after the addition of HMF, resulting in degradation of the furan [17]. In addition to redundant and/or unique functions, it is also possible that some more complex, synergistic, or contradicting interactions between SSPs will eventually be found. In the case of effectors, which have been studied in pathogenic interactions in more depth than saprophytic SSPs, Arroyo-Velez et al. [34] suggested that functions achieved by the repertoire of proteins are more than the additive effects of the individual effectors. Whether the effects observed here are directly due to the changes imposed on the expression of SSPs or mediated by additional genes/proteins in *P. ostreatus* has not yet been resolved. Thus, while we have demonstrated the differential involvement of specific SSPs in substrate utilization by a saprophyte, the actual mechanistic nature of SSP functions remains to be determined. 

While only a partial reduction in *ssp4* transcription, along with an increase in *ssp6* transcript abundance, were evident in the KD*ssp1* strain, this was accompanied by a significant and specific reduction in the efficacy of lignin removal, indicative of the relevance of these genes to lignin degradation. This was further substantiated by the fact that *vp2* and *mnp1* transcript levels were also reduced in this strain. The apparently coordinated regulation of expression observed here is probably not limited to the transcript level, as VP2 and MnP1 proteins were shown to accumulate in *P. ostreatus* growing on a woody substrate [35]. *vp2* transcript was also shown to be abundant in the wild-type strain of *P. ostreatus* grown on toluene/ethanol-extracted beechwood sawdust for 13 days but was significantly reduced in all mutant strains exhibiting reduced lignin-degrading abilities on that substrate [20,32]. In a more recent study, Wu et al. [19] reported that *vp2* expression was significantly decreased (about one-thousandth) in a *hir1* (histone chaperone protein) disruptant that exhibited reduced lignin-degrading capacity relative to the wild type. Under those conditions, *mnp1* expression was not affected, while *vp1* expression was increased. In contrast, the expression of *mnp3* was significantly downregulated on beechwood sawdust, a change that was not observed in the current study on the wheat straw substrate. In the current study, lignin degradation was not completely attenuated as a result of the reduction in the expression of two dominant ligninolytic peroxidases, *vp2* and *mnp1.* This may be due to the compensatory effects of other components of the lignin-degrading machinery (e.g., *mnp2*; [36]) that were either unaffected or even exhibited increased expression (e.g., *mnp5*). Considering the recently proven redundancy of some components of the lignin-degrading machinery [37], it is likely that these are preferentially involved in lignin degradation under varying circumstances. Recently, using multiple *mnp*/*vp*/*lac* mutants of *P. ostreatus,* Nakazawa et al. [38] demonstrated that MnPs and VPs have overlapping roles in the degradation of natural lignin.

When considering the transcriptional response of *P. ostreatus* to different substrates, ranging from liquid media to straw and woody substrates, the findings described by Wu et al., [19] are strongly substantiated by our current study. In their study, in the context of impaired lignin degradation, the fungus expressed variable, substrate-dependent gene and protein repertoires involving distinct lignocellulolytic genes. At the same time that deletion of genes like *gat1 (*a putative transcriptional regulator) and *pex1* (encodes a peroxisome biogenesis factor) affect the lignin degradation machinery, their inactivation can also affect the transcription of additional, apparently unrelated, genes. Pectinases are one such example. In our study, *ssp* silencing resulted in an increase in pectin lyase gene expression on the wheat straw medium, which was also evident as increased enzymatic activity when the strain was cultured on a pectin-rich medium (Figure 6). In contrast, Wu et al. [32] demonstrated an opposite phenomenon in the two above-mentioned mutants, Δ*gat1* and Δ*pex1*. In addition, they observed a difference in the expression of pectinases in the *P. ostreatus* wild-type strains when comparing two substrates, rice straw and beechwood sawdust, suggesting that the fungus’ specific response to wheat straw may play a significant factor in the genetic and physiological response. 

In addition to modulating part of the lignin-degrading machinery, this study has also provided evidence for a possible link between *ssp*s and other genes with possible functions related to lignin degradation (Table 1). One of these is *hemopexin*, (id 133592), which encodes for a protein that exhibits hemin-binding properties and has been designated *ostreopexin* in *P. ostreatus* [39]. While its role in lignin degradation has not been investigated, it is conceivable that it may be involved in transferring heme to ligninolytic peroxidases in a similar manner as hemin and hemoglobin [40,41]. Alternatively, it may serve as an ROS scavenger involved in protecting the cell from oxidative damage [42]. Another example encompasses the hydrophobins. The transcript levels of one hydrophobin, 74127, was upregulated by 3.6-fold in the KD*ssp1* mutant, which was similar to that observed in the lignin degradation-impaired mutant Δ*hir1* [19]. Interestingly, an opposite expression pattern of this hydrophobin was observed in a different lignin degradation-impaired mutant—Δ*gat1* [19]. Another hydrophobin-encoding gene, 63361, exhibited reduced expression levels in the KD*ssp1* strain, which is in contrast to that previously reported by Wu et al. [32]. These results emphasize the complexity of the regulation and expression patterns of some genes, such as hemopexin and hydrophobins (and other genes whose functions vis-à-vis lignin degradation are not known) in different genetic backgrounds and as affected by the specific growth substrate.

In addition to the variations in the genetic backgrounds and the use of different lignocellulosic substrates in the abovementioned studies, the fact that most of them were based on a single time point sampling should be taken into consideration. One of the results of the current study points to the fact that the most significant differences observed in the transcriptional profiles were between two and three weeks of culturing on the straw substrate. This is highly indicative of an adaptive, rather than inductive, response of the fungus to the growth niche. This concept, shown here along the three-week sampling period, is in high agreement with the recent analysis described by Nakazawa et al. [38] and warrants a future emphasis on deciphering the kinetics of the substrate utilization process. In addition to the marked temporal changes observed in the measured transcriptional profiles, further analyses using WGCNA [43], which has been previously used to identify genes associated with sporulation processes in *Ganoderma lingzhi* [44] and for discovering the landmark genes involved in mushroom development and the evolution of complex multicellularity [45], we were able to identify key modules of genes highly associated with the *ssp* knock-down genotype (Figure 7 and Figure 8). This, in turn, allowed us to provide a genome-scale overview of bioprocesses that may be directly or indirectly modulated by some *ssp*s in *P. ostreatus*. Similar to the analysis of DEGs, scrutiny of the modules revealed potential positive, as well as negative, gene-expression modulation by SSPs. One example involves functions related to pectin and side-chain hemicellulose degradation, which we were able to functionally verify in culture. At the same time, the enhanced expression of genes associated with mRNA processing and ribosomal synthesis, which occurred in the KD*ssp1* strain, could be indicative of the basic need for increased synthesis of proteins in the mutant. This response is consistent with our previous findings suggesting regulatory roles of SSPs in the transition between the trophophase and idiophase growth phases of the fungus [21]. Nonetheless, the possible presence of a functional link between the involvement of SSPs in developmental transitions and modulation of the lignocellulosic machinery has yet to be elucidated. 

The results of this study have provided evidence for the differential roles members of the structurally related *ssp1-6* gene family likely play in lignocellulosic substrate utilization by *P. ostreatus*, as represented by the dynamics of the fungus’ transcriptional profiles during growth on a natural wheat straw substrate. Further functional dissection of the identified processes is likely to provide additional, much-needed, mechanistic insight as to the function of saprophytic SSPs and may also enhance our capabilities of manipulating *P. ostreatus* and other WRF for improved residual plant biomass conversion processes.

## 4. Materials and Methods

### 4.1. Fungal Growth and Experimental Conditions

The wild-type *P. ostreatus* var *ostreatus* (Jacq.) P. Kumm. monokaryotic strain PC9 (Spanish Type Culture Collection (CECT) accession number CECT20311), which is a protoclone derived by de-dikaryotization of the commercial dikaryotic strain N001 (CECT20600) [46] (Larraya et al., 1999), was used throughout this study. The RNAi KD*ssp1* strain was generated during a previous study on the basis of RNAi expression [17,47], and its genetic nature was re-verified prior to its utilization. The strains were grown and maintained on either Potato Dextrose Agar (PDA, Difco, Franklin Lakes, NJ, USA), cultured for in vitro *ssp* gene-induction experiments on Glucose Peptone (GP) medium (20 g L^−1^ glucose, 5 g L^−1^ peptone, 2 g L^−1^ yeast extract, 1 g L^−1^ K_2_HPO_4_, and 0.5 g L^−1^ MgSO_4_·7H_2_O), or for straw innoculum preparation on Basidiomycete Salt Medium (BSM) (5 g L^−1^ glucose, 1 g L^−1^ K_2_HPO_4_, 0.6 g L^−1^ asparagine, 0.1 g L^−1^ yeast extract, 0.5 g L^−1^ KCl, 0.5 g L^−1^ MgSO_4_·7H_2_O, 3 mg L^−1^ Zn(NO_3_)_2_·6H_2_O, 6 mg L^−1^ Ca(NO_3_)_2_·4H_2_O, and 3 mg L^−1^ CuSO_4_·5H_2_O) [1]. When required, 15 g L^−1^ agar was added to the medium. For growth experiments on the natural substrate, 8 agar discs (3-mm) of the fungal mycelia that had been cultured on BSM were evenly placed on 5 separate, moist, sterilized wheat straw substrate plates (2 g straw with 7 mL of water per 90 mm glass Petri dish) per treatment. Wheat straw was autoclaved twice on consecutive days prior to use. Cultures were maintained at 25 °C in the dark.

### 4.2. Nucleic Acid Isolation and Gene Expression Analyses

Total RNA was extracted from culture biomass, which was first flash frozen with liquid nitrogen, ground with mortar and pestle, and subsequently extracted using the Spectrum Plant Total RNA Kit (Sigma, St. Louis, MI, USA) with a modification that included an increase in the sample size (500 mg) and lysis buffer used (750 µL) per replicate. Samples from each of the five inoculated dishes per treatment were independently extracted. To remove DNA contamination from total RNA, on-column DNase treatment was carried out using the Qiagen RNase-Free DNase Set kit and Qiagen Mini RNeasy™ kit (Qiagen, Germantown, MD, USA). RNA quality was analyzed via RNA gel electrophoresis and an RNA ScreenTape assay on an Agilent 2200 TapeStation system (Agilent Technologies, Santa Clara, CA, USA) to validate RNA quality. Only samples exhibiting a RIN number > 8.0 were used for cDNA synthesis. Plate-based RNA sample (5 replicates of each treatment) prep was performed on the PerkinElmer Sciclone NGS robotic liquid handling system using Illumina’s TruSeq Stranded mRNA HT sample prep kit. This utilized poly-A selection of mRNA while following the protocol outlined by Illumina in their user guide, https://support.illumina.com/sequencing/sequencing_kits/truseq-stranded-mrna.html, accessed on 1 March 2023, with the following conditions: total RNA starting material was 1 µg per sample, and 8 cycles of PCR were used for library amplification. The prepared libraries were then quantified using KAPA Illumina library quantification kit (Roche, Basel, Switzerland) and run on a LightCycler 480 real-time PCR instrument (Roche). The quantified libraries were then multiplexed, and the pool of libraries was then prepared for sequencing on the Illumina NovaSeq 6000 sequencing platform using NovaSeq XP v1.5 reagent kits (Illumina, San Diego, CA, USA), S4 flow cell, following a 2 × 150 indexed run recipe.

### 4.3. RNA-Seq Data Processing and DEGs

For RNA-seq data processing, raw fastq file reads were filtered and trimmed using the JGI (DOE Joint Genome Institute) QC pipeline. Using BBDuk (https://sourceforge.net/projects/bbmap/; accessed on 1 March 2023), raw reads were evaluated for artifact sequence by kmer matching (kmer = 25), allowing 1 mismatch, and detected artifact was trimmed from the 3’ end of the reads. RNA spike-in reads, PhiX reads, and reads containing any Ns were removed. Quality trimming was performed using the phred trimming method set at Q6. Following trimming, reads under the length threshold were removed (minimum length of 25 bases or 1/3 of the original read length—whichever was longer). Filtered reads from each library were aligned to the reference genome of *P. ostreatus* PC9 v1.0 (http://genome.jgi.doe.gov/PleosPC9_1/PleosPC9_1.info.html; accessed on 1 March 2023) using HISAT2 version 2.2.0 [48]. Strand-specific coverage bigWig files (fwd and rev) were generated using deepTools v3.1 [49]. FeatureCounts [50] was used to generate the raw gene count files using gff3 annotations. Only primary hits assigned to the reverse strand were included in the raw gene counts (-s 2 -p --primary options). Raw gene counts were used to evaluate the level of correlation between biological replicates using Pearson’s correlation, shown in heatmap, and determine which replicates would be used in the DGE analysis. The values of FPKM (Fragments Per Kilobase of transcript per Million mapped reads) were calculated to present genes’ expression levels.

DESeq2 (version 1.30.0) [51] was used to determine the differentially expressed genes (DEGs) between pairs of conditions. The parameters used to call a DEG between conditions were adjusted *p*-value < 0.05 (i.e., FDR < 0.05). Fold changes of genes were calculated using FPKM values. Individual results for each pairwise comparison are in the directory Pairwise DGE Results (Table S1). 

### 4.4. Co-Expression Network Analysis

Co-expression patterns of genes were analyzed by Weighted Correlation Network Analysis (WGCNA; [29]). Constitutively lowly expressed genes were removed; thus, 9742 out of 12204 whole genes with expression levels of FPKM > 1 in all samples were kept for the network analysis. Sample tree was built using the “average” method to cluster samples and determine the outliers. Adjacency correlation matrix was calculated with softPower of 25 for “signed” networks, followed by gene tree construction and module identification. The merged modules of genes were then generated along with eigengenes to analyze their relevance to temporal stages and genotypes, the hub genes with the function of “chooseTopHubInEachModule”, and the gene ontology (GO) functional enrichment.

### 4.5. Functional Analysis of the Co-Regulated Modules

Functional annotation by OmicsBox v3.0.29 was performed to reannotate genes’ functions of the *P. ostreatus* PC9 v1.0 genome, as previously described [52]. The most recently updated (till 20 March 2023) protein database was incorporated into the analyses. Secreted proteins were predicted by SignalP_no^TM^ domain by using SignalP6.0 [53]. GO enrichment was conducted for DEGs and gene modules resolved by WGCNA.

### 4.6. Cell Wall Component Analyses

Determination of wheat straw substrate cell wall composition was performed following the sequential method of Van Soest et al. [24] with the adapted protocol of Ankom Technology (Macedon, NY, USA). Briefly, two grams of non-inoculated substrate and solid-state fermentation products of the PC9 and KD*ssp1* mutant that were harvested at 3 time points (7, 14, and 21 days) were lyophilized, ground to pass a 1 mm pore-size screen in a Wiley mill, and placed in Ankom fiber filter bags (F57; Macedon, NY, USA). First, the neutral detergent fiber (NDF) was used, followed by digestion with acid detergent solution to determine the acid detergent fiber (ADF) representing cellulose and lignin. The acid detergent lignin (ADL) content was determined gravimetrically as the residue remaining upon ignition at 600 °C after treatment with 72% H_2_SO_4_ to remove cellulose. Hemicellulose was calculated as NDF—ADF. All fractions were expressed on a dry matter basis.

### 4.7. Pectin Degradation

The capacity of the fungal strains to degrade pectin was determined by visualizing the pectin degradation halo visualized using a Congo Red staining protocol [54]. Pectin agar medium (PAM) was prepared according to Haile et al. [54] and was composed of ammonium sulfate, 2 g L^−1^; yeast extract, 1 g L^−1^; Na_2_HPO_4_, 6 g L^−1^; KH_2_PO_4_, 3 g L^−1^; apple pectin (Sigma, USA), 5 g L^−1^; and agar, 20 g L^−1^, pH 5.5 ± 0.5 (adjusted using 6M HCl). Then, 5 mm fungal plugs were placed in the center of pectin agar media plates in triplicate and incubated for 5–14 days at 25 °C in the dark. After the incubation period, the surface of the culture medium was covered with 0.3% Congo Red solution, incubated at room temperature for 15 min, and then washed with double-distilled water. Clear zones (halos) evident around the colonies provided an indication for extracellular pectinase activity. The diameter of the halo has been shown to have a direct relationship with the relative pectinase production capacity [55].

## Figures and Tables

**Table 1:** Significant DEGs in the KD*ssp1* strain versus the PC9 wild type over a three-week period of growth on a wheat straw substrate.

Protein IDs ^a^	Weighted Gene Co-Expression Network Analysis (WGCNA) Modules ^b^	Biological Description ^c^	SP ^d^	Fold Change of DEGs ^e^		
				7 d	14 d	21 d
**Genes downregulated in KD mutants**						
133592	darkgreen	Hemopexin-like protein	N	1.14	492.00	46.47
86351	darkgreen	Hypothetical self-proteolytic membrane RHS protein	N	1.16	14.00	22.30
117678	darkgreen	Troponin-like	N	1.01	11.43	20.20
108294	darkgreen	3′-5′ Exonuclease	N	1.38	10.09	15.92
122257	darkgreen	Unknown protein	N	2.45	11.17	10.01
89221	darkgreen	Membrane attack complex component/perforin domain	N	5.25	7.88	8.16
113854	darkgreen	Unknown protein	N	16.64	13.98	8.14
116044	darkgreen	O-methyltransferase	N	3.73 ^e^	18.79	7.92
87569	darkgreen	Unknown protein	N	1.07 ^f^	7.37	7.74
72169	darkgreen	Unknown membrane protein	Y	15.75	14.07	7.27
123506	orangered4	Unknown protein	N	0.48 ^f^	4.20	7.16
117582	darkgreen	Voltage-gated shaker-like K+ channel, subunit beta/KCNAB	N	8.02	15.53	7.11
122258	darkgreen	Hypothetical helicase	N	1.87	4.86	6.80
101257	darkgreen	Aldo/keto reductase	N	2.70 ^f^	14.77	6.33
87540	orangered4	Unknown protein	N	2.99 ^f^	16.94	6.15
88910	darkgreen	GMC oxidoreductase	N	1.16 ^f^	4.48	5.91
39792	darkgreen	Hypothetical velvet factor	N	1.24	5.26	5.71
60432	darkgreen	Versatile peroxidase; AA2	Y	8.82	4.58	5.66
79338	darkgreen	Nitronate monooxygenase	Y	1.20	5.00	5.58
130698	darkgreen	Unknown membrane protein	N	3.92	4.82	5.39
81199	darkgreen	Glucose/ribitol dehydrogenase	Y	5.73 ^f^	6.65	5.06
46715	darkgreen	SKP1/BTB/POZ domain containing	N	2.93	5.59	4.98
126430	darkgreen	Hypothetical isomerase	N	3.37	6.20	4.76
113642	darkgreen	Hypothetical zinc finger protein	Y	1.07	5.64	4.71
117317	darkgreen	Unknown protein	Y	2.70	4.87	4.56
99670	darkgreen	Galactose oxidase-like, AA5	Y	0.82 ^f^	5.36	4.49
87426	darkgreen	Asl1-like glycosyl hydrolase	Y	2.20	6.53	4.30
**Genes upregulated in KD mutants**						
89942	coral1	alpha-galactosidase, GH27	Y	0.74 ^f^	11.96	32.01
117545	darkslateblue	Lysozyme, GH25	Y	1.98	5.93	28.01
83989	coral1	Pectin lyase	Y	0.55 ^f^	8.57	24.72
44086	coral1	Hypothetical cerato-platanin protein	Y	0.47 ^f^	9.57	19.44
96680	darkslateblue	Lysozyme, GH24	Y	1.28 ^f^	5.36	12.85
132186	black	Hypothetical cerato-platanin protein	N	2.17 ^f^	6.01	12.68
110536	coral1	Hypothetical short-chain dehydrogenase	Y	1.22 ^f^	4.03	12.41
104355	darkslateblue	Proline-rich protein	Y	4.16	10.74	12.03
97623	coral1	alpha-N-arabinofuranosidase, GH43	Y	0.57 ^f^	5.53	11.86
51760	darkslateblue	Endo-polygalacturonase, GH28	Y	0.83 ^f^	4.33	11.84
87572	darkslateblue	Hypothetical cupredoxin protein	Y	1.34 ^f^	11.25	11.60
56468	darkslateblue	Sugar transporter	Y	1.87	7.31	11.02
55434	coral1	Pectate lyase	Y	0.54 ^f^	4.67	10.74
87101	darkslateblue	Cytochrome P450 CYP2 subfamily	Y	1.82	11.82	10.52
59334	darkslateblue	Pectin lyase, GH28	Y	1.34 ^f^	5.55	10.07
99620	coral1	Protein-tyrosine-phosphatase	N	0.73 ^f^	6.09	10.01
98979	darkslateblue	Unknown protein	N	2.26	4.55	8.25
31260	darkslateblue	Hypothetical membrane protein	Y	2.98	6.68	6.81
126905	coral1	alpha-L-rhamnosidase, GH37	N	1.08 ^f^	6.12	6.74
88830	black	Hypothetical membrane protein	Y	6.99	10.23	6.60
65815	black	Carbohydrate-Binding Module Family 13	Y	1.48	5.12	6.58
61773	coral1	Lipase_GDSL, CE16	Y	1.14	4.37	6.56
116339	darkslateblue	Carotenoid ester lipase precursor	Y	2.49	4.41	5.46
126566	darkslateblue	Carotenoid ester lipase precursor	N	1.96	6.52	5.46
91336	darkslateblue	Unknown protein	N	3.35	5.08	4.89
62766	darkslateblue	GH32	Y	1.87	4.24	4.86
95957	darkslateblue	Hypothetial dienelactone hydrolase	N	1.38	4.58	4.83
117665	black	Unknown protein	Y	1.25	5.90	4.76
131254	darkslateblue	Chromo (CHRromatin Organisation MOdifier) domain	N	2.86	4.67	4.58
114119	darkslateblue	Nitronate monooxygenase	N	2.39	4.47	4.18

^a^ Protein IDs were retrieved from the genome annotation of *Pleurotus ostreatus* PC9 v1.0 at JGI database (https://mycocosm.jgi.doe.gov/PleosPC9_1/PleosPC9_1.home.html; accessed on 1 March 2023). ^b^ DEGs were assigned to the specific gene modules obtained by WGCNA network analysis as in the following section. ^c^ Functional annotation was performed with OmicsBox v3.0.29 and then confirmed by searching the mostly updated InterPro database. ^d^ Existence of protein secretion signals predicted by SignalP 6.0, with “Y” for yes and “N” for no. ^e^ DEGs were selected by using the fold change cut values of 4 for pairwise comparisons for each time point and were ranked based on fold changes of the 21-day period. ^f^ Genes that have FDR values of more than 0.05 and were omitted from the overall DEG analysis were highlighted for the 7-day pairwise comparisons.

## Data Availability

RNA-seq data generated in this work were deposited at the Sequence Read Archive (SRA) with the BioProject accession numbers PRJNA978702-978660.

## Supplementary Materials

The supporting information can be downloaded at: https://www.mdpi.com/article/10.3390/ijms242316828/s1.
