# Supplementary material for: Altered Expression of Two Small Secreted Proteins (ssp4 and ssp6) Affects the Degradation of a Natural Lignocellulosic Substrate by Pleurotus ostreatus
Source: Int J Mol Sci. 2023 Nov 27;24(23):16828. doi: 10.3390/ijms242316828 (PMC10705924; doi:10.3390/ijms242316828)
Supplement: Supplementary file 1 [file ijms-24-16828-s001.zip › ijms-2724555-supplementary/Figure S1.PPTX]

## Slide 1
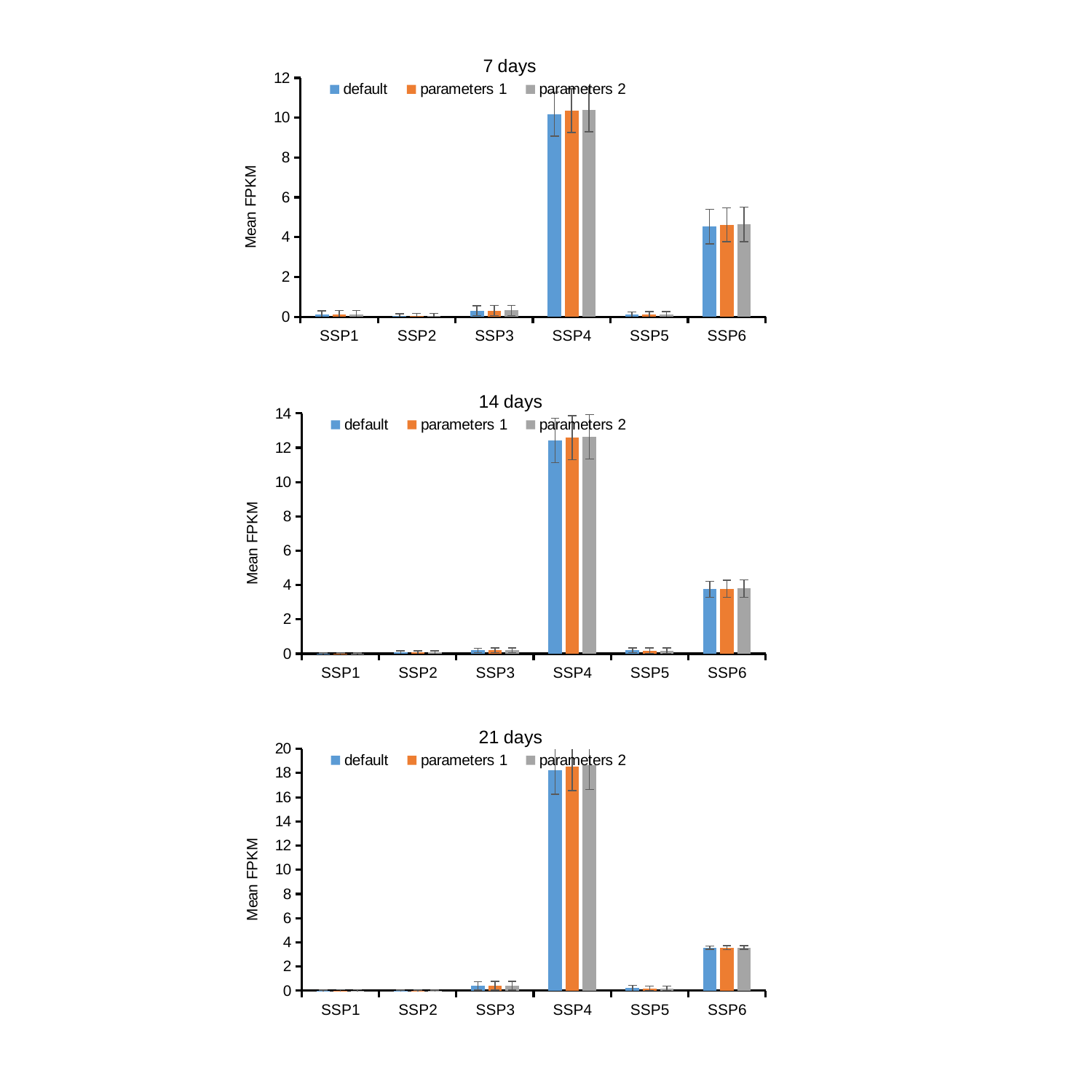

### Chart: 7 days
| Category | default | parameters 1 | parameters 2 |
|---|---|---|---|
| SSP1 | 0.122 | 0.126 | 0.126 |
| SSP2 | 0.052000000000000005 | 0.054000000000000006 | 0.054000000000000006 |
| SSP3 | 0.30399999999999994 | 0.312 | 0.31399999999999995 |
| SSP4 | 10.176 | 10.348 | 10.4 |
| SSP5 | 0.122 | 0.124 | 0.124 |
| SSP6 | 4.526 | 4.618 | 4.64 |
### Chart: 14 days
| Category | default | parameters 1 | parameters 2 |
|---|---|---|---|
| SSP1 | 0.016 | 0.018 | 0.018 |
| SSP2 | 0.074 | 0.074 | 0.076 |
| SSP3 | 0.198 | 0.202 | 0.202 |
| SSP4 | 12.423999999999998 | 12.582 | 12.638 |
| SSP5 | 0.20000000000000004 | 0.18800000000000003 | 0.19 |
| SSP6 | 3.7600000000000002 | 3.786 | 3.8019999999999996 |
### Chart: 21 days
| Category | default | parameters 1 | parameters 2 |
|---|---|---|---|
| SSP1 | 0.022 | 0.024 | 0.024 |
| SSP2 | 0.018 | 0.02 | 0.02 |
| SSP3 | 0.394 | 0.404 | 0.40800000000000003 |
| SSP4 | 18.244 | 18.544 | 18.627999999999997 |
| SSP5 | 0.21200000000000002 | 0.194 | 0.194 |
| SSP6 | 3.5520000000000005 | 3.554 | 3.572 |
