# Supplementary material for: Altered Expression of Two Small Secreted Proteins (ssp4 and ssp6) Affects the Degradation of a Natural Lignocellulosic Substrate by Pleurotus ostreatus
Source: Int J Mol Sci. 2023 Nov 27;24(23):16828. doi: 10.3390/ijms242316828 (PMC10705924; doi:10.3390/ijms242316828)
Supplement: Supplementary file 1 [file ijms-24-16828-s001.zip › ijms-2724555-supplementary/Table S2.docx]

**Table S2.** Percent identity of *ssp* gene family members to that present in the RNAi construct used to reduce *ssp* gene family expression.

| **gene** | **Protein ID** | **Presence of continuous ≥ 18 bp sequence identity** |
| --- | --- | --- |
| *ssp1* | 65712 | Yes (160bp) |
| *ssp2* | 46202 | Yes (35bp) |
| *ssp3* | 91630 | Yes (35bp) |
| *ssp4* | 90499 | Yes (19bp) |
| *ssp5* | 44261 | Yes (20bp) |
| *ssp6* | 100993 | No (11bp) |
